# An investigation on humans’ sensitivity to environmental temperature

**DOI:** 10.1038/s41598-023-47880-5

**Published:** 2023-12-04

**Authors:** Laura Battistel, Andrea Vilardi, Massimiliano Zampini, Riccardo Parin

**Affiliations:** 1 Center for Mind/Brain Sciences (CIMeC), University of Trento, Corso Bettini 31, Rovereto, TN Italy; 2 terraXcube, Eurac Research, Via Ipazia 2, 39100 Bolzano, Italy

**Keywords:** Psychology, Psychology and behaviour, Cognitive neuroscience, Sensory processing

## Abstract

While earlier investigations into thermal perception focused on measuring the detection of temperature changes across distinct bodily regions, the complex nature of thermal perception throughout the entire body remains a subject of ongoing exploration. To address this, we performed an experiment using four climate chambers with oscillating temperatures between 24 °C ± 1 °C. Our study involved 26 participants who moved between these chambers and had the task of reporting whether the second chamber entered was warmer or colder than the previous one. We collected 3120 temperature judgments, which we analysed via generalised linear mixed-effects models. The results showed surprisingly accurate temperature discrimination abilities and limited variation between individuals. Specifically, the Point of Subjective Equality stood at − 0.13 °C (± 0.02 °C), the Just Noticeable Difference (JND) was 0.38 °C (± 0.02 °C), the JND^95^ (indicating 95% accuracy) 0.92 °C (± 0.05 °C), the negative ceiling performance level (CPL) was − 0.91 °C (± 0.28 °C) and the positive CPL 0.80 °C (± 0.34 °C). The implications of the JND^95^ and the CPLs are particularly noteworthy, as they hold potential to significantly contribute to the advancement of intelligent algorithms for temperature control systems within building environments.

## Introduction

As humans, our perception and understanding of the world are shaped by the unique characteristics and stimuli present in the environment. Various psychological theories attempt to explain how our mind integrates this information to form a unified and comprehensive worldview. According to the grounded cognition theory^1^, the process of perception and cognition does not involve combining the different sensory modalities to form a final semantic representation that transcends individual senses. Instead, it emphasises the connection between our minds, bodies, and the environment. Even during abstract reasoning, our thoughts are grounded in our bodily experiences and activate sensorimotor memories^1^. Therefore, it may be worthwhile to investigate how our environment affects our minds.

The environment encompasses various factors that can impact our cognition, including green spaces, humidity levels, natural light, and temperature^2^. Temperature, in particular, plays a crucial role in our survival, as the maintenance of a constant body and brain temperature is necessary for proper physiological functioning^3^. To perceive temperature, our bodies possess specialised sensory receptors called thermoreceptors. These thermoreceptors are free nerve endings located in the skin, which transmit signals to the spinal cord and thalamus via the thermosensory spinothalamic tract^4^. Interestingly, different thermoreceptors are responsible for processing cold and warm stimuli, indicating partial segregation of these processes^4^. Furthermore, thermoreceptors exhibit distinct responses to static and dynamic temperature stimuli. When entering a warm environment, warm thermoreceptors become more active compared to cold thermoreceptors. Conversely, when the environment starts to cool down, the firing rate of cold thermoreceptors suddenly increases. As the environment becomes cold, cold thermoreceptors continue to respond, while the activity of warm thermoreceptors decreases^5^. This dynamic behaviour enables us to be aware of the current temperature we are exposed to and any potential temperature fluctuations in the environment.

The thermosensory spinothalamic tract subsequently reaches the posterior insular cortex, which serves as the primary thermosensory cortex involved in both discriminatory and affective processes associated with skin thermal sensations^6^. This cerebral area is also responsible for the broader representation of our body, known as interoception^4^. The signals originating from this brain region play a fundamental role in maintaining homeostasis and regulating the body’s internal state. Thermoregulation encompasses various mechanisms, including autonomic reflexes (such as shivering or sweating)^7^ and conscious behaviours (i.e., change of clothing) that we have learned to employ in order to feel comfortable even when the environment is changing^8^. To effectively perform these adaptive behaviours, we also need to perceive the thermal proprieties of one own’s body and the surrounding environment. Research suggests that the thermal sensations coming from the head play a crucial role in our perception of thermal comfort^9^, and changes in peripheral temperature have a more significant impact on our thermal regulation compared to changes in core temperature^10^.

Previous research has predominantly focused on quantifying human temperature sensitivity by applying thermal stimuli to specific areas of the body. These studies have identified the specific temperature ranges with distinct thermal perceptions: the range perceived as extremely cold or hot (< 15 °C/> 45 °C) and that interpreted as comfortably cool or warm (15 °C < T < 30 °C / 30 °C < T < 45 °C)^11–14^. In practice, the thermal stimulus applied to participants typically involves a small thermode—a compact metal plate capable of controlled cooling and heating. Furthermore, this research has revealed varying thermal sensitivities across different body parts^15–18^, with the head being more sensitive to warm temperatures and the torso more responsive to cold stimuli^19^. In a recent study, Crucianelli et al. (2021) found that the minimum difference participants could perceive (also known as the just noticeable difference, JND) in cold stimuli applied to the palm is 2.02 °C, while is 1.48 °C on the forearm. For warm stimuli, the JND on the palm is 2.65 °C, and on the forearm, is 2.61 °C^20^.

**Figure 1:**
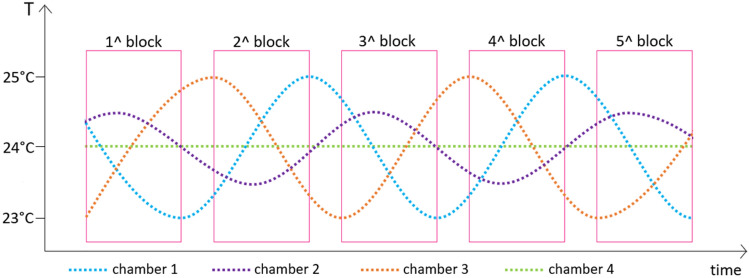
Temperature patterns imposed to the four climate chambers.

**Figure 2:**
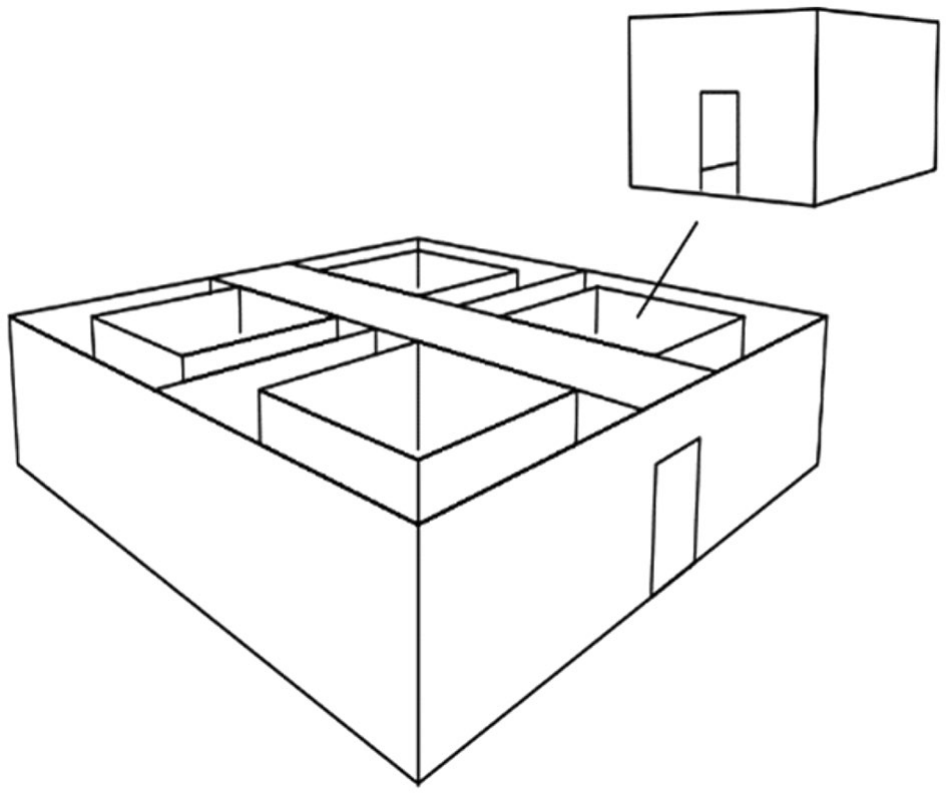
3D model of the laboratory: four climate chambers and airlock are displayed.

However, a notable gap in the existing literature pertains to the JND when individuals are immersed in a specific temperature environment, involving their entire body in the perceptual process. To address this knowledge void, we conducted a study specifically aimed at determining human sensitivity to a comfortable environmental temperature of 24 °C ± 1 °C (Fig. 1). Participants were instructed to move between four distinct climate chambers (Fig. 2), each presenting different temperature levels, and were tasked with comparing the temperatures in each chamber to discern the minimum difference they could perceive (i.e., the JND). By focusing on thermal sensation in the context of a holistic body experience within a comfortable environment, our study aims to shed light on a crucial aspect of temperature perception that has yet to be thoroughly explored. The insights gained from this investigation hold potential implications for various domains, such as thermal comfort regulation in real-life settings and the design of climate-controlled environments that account for human sensitivity to temperature changes.

## Results

Table 1 summarises the demographical information of the 26 participants. On average, they have a normal emphatic quotient and normal awareness of their internal bodily states, and they have a normal level of daily activity. Regarding their usual preferences for temperature, 13 of them declared to prefer a cold environment and to get easily warm during different activities; 4 participants reported the opposite, so they prefer warm environments and get easily cold; and the remaining 9 participants did not have clear preferences.

**Table 1:** Summary of 26 participants’ demographical information.

26 participants	Age	Sex	BMI	Place of birth	Temperature preferences	EQ	BPQ_BOA	BPQ_ASR	GPAQ_m	GPAQ_i
characterisation	max 47; min 20	50% females	max 24.8; min 19	18 born in north Italy, 7 born in south Italy	4 prefer warm, 9 no preferences, 13 prefer cold	5 < cut-off	8 < cut-off	1 > cut-off	1 < cut-off	6 < cut-off
Average	30	–	22	–	–	41	21	15	600 [min]	382 [min]
St. deviation	6	–	2	–	–	8	6	2	468 [min]	460 [min]

*EQ* Emphatic Quotient (cut-off < 33), *BPQ_BOA* Body Perception Questionnaire_Body Awareness (cut-off < 18), *BPQ_ASR* Body Perception Questionnaire_Autonomous System Stress Response (cut-off > 16), *GPAQ_m* Global Activity Questionnaire_moderate level of activity (cut-off < 150 min), *GPAQ_i *Global Activity Questionnaire_intense level of activity (cut-off < 75 min).

As described in the analysis section, we first proceeded in computing the two generalised linear mixed-effects models (glmm0 and glmm1) and then we compared their goodness of fit by means of an ANOVA. The results are reported in Table 2. The Akaike information criterion (AIC) of the model considering also the difference in temperature as a random effect (glmm1) is smaller, indicating that it explains better our data with respect to the model considering only the participants as a random effect. The same information is extracted by looking at the Bayesian information criterion (BIC) and the significant *p* value.

**Table 2:** Results of the ANOVA between the two generalised linear mixed-effects models.

Models	AIC	BIC	Pr(> Chisq)
glmm0	2389.2	2407.3	
glmm1	2363.0	2393.3	2.85e−07 ***

*glmm0* model considering only participants as random effects, *glmm1* model considering both the temperature differences and the participants as random effects, *AIC* Akaike information criterion, *BIC* Bayesian information criterion, Pr(> Chisq) = *p* value.

Next, we calculated the R^2^ of glmm1 to analyse the percentage of variance explained by the fixed effects alone (R2m) and by the fixed effects together with the random effects (R2c). Our fixed effects alone explain 68% of our data, while the addition of the random effect added 4% of the explanation (see Table 3). Taken together, these results show both the adequacy of our model in explaining our data and the small variability that the single participants add to them.

**Table 3:** Results of the R^2^ calculation for glmm1.

R2m	R2c
0.68	0.72

*R2m* marginal R-squared, *R2c* conditional R-squared.

The summary of the model (Table 4) shows that for each increment of ± 1 °C, the probability to have a correct answer is almost double (Estimate = 1.79) and therefore the participants’ sensitivity drastically increases.

**Table 4:** Summary of the model glmm1.

Fixed Effects	Estimate	Std. Error	z value	Pr( >|z|)
(Intercept)	0.23	0.04	5.30	1.16e−07 ***
DT [°C]	1.79	0.10	17.82	< 2e−16 ***

*DT [°C]* temperature differences, Pr( >|z|) = *p* value.

**Figure 3:**
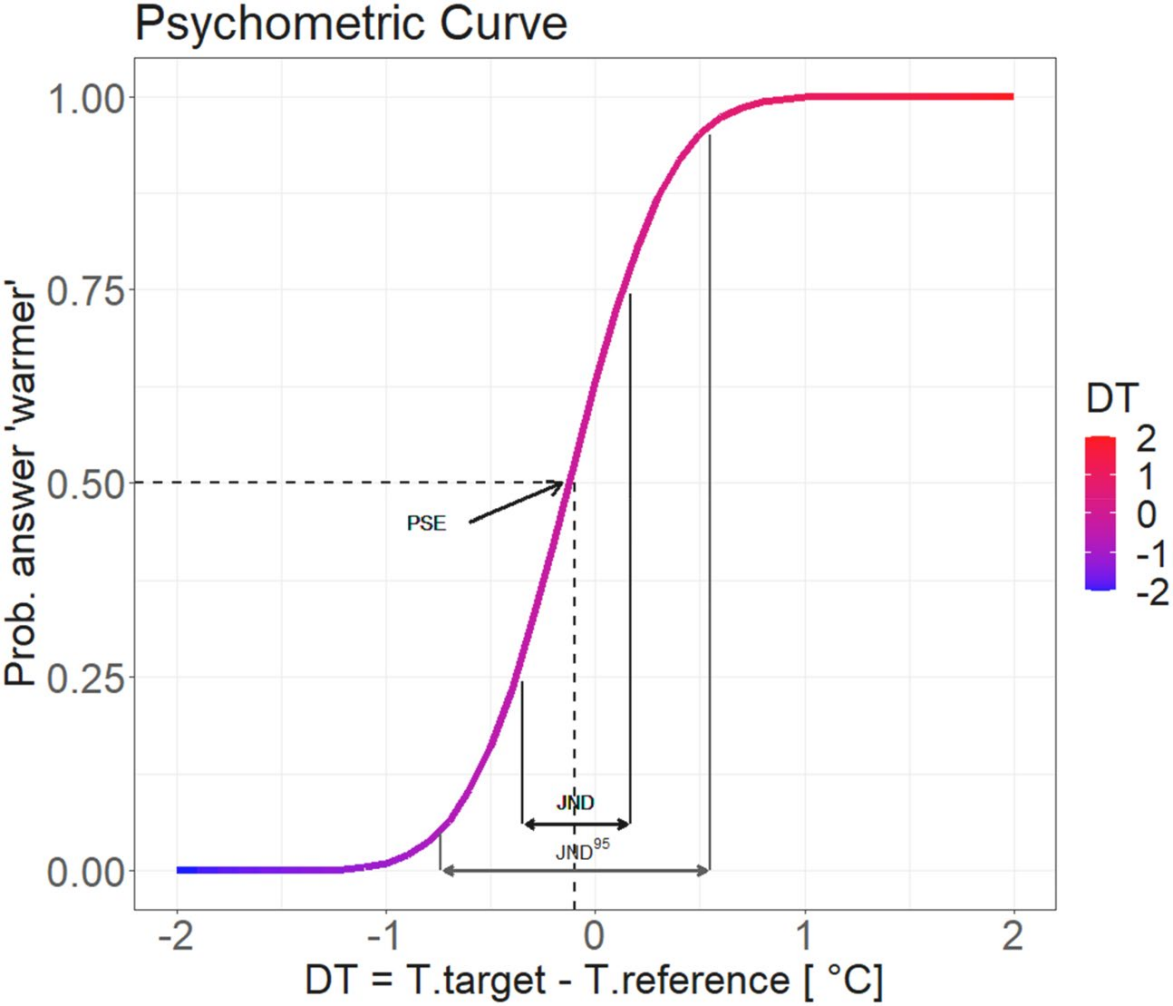
Psychometric function averaged on the 26 participants.

Then, we calculated the average PSE, the JND and the JND^95^ (i.e., representing 95% of accuracy) using the function MixDelta. Table 5 reports these results showing a PSE of − 0.13 °C, a JND of 0.38 °C and a JND^95^ of 0.92 °C, while Fig. 3 shows the average psychometric curve obtained with these data.

**Table 5:** PSE and JND results.

	Estimate (°C)	Std. error (°C)	Inferior (°C)	superior (°C)
PSE	− 0.13	0.02	− 0.17	− 0.08
JND	0.38	0.02	0.33	0.42
JND^95^	0.92	0.05	0.82	1.02

*PSE* Point of subjective equality, *JND* Just noticeable difference (representing 75% of accuracy), *JND95* JND representing 95% of accuracy.

The ceiling performance levels were + 0.8 °C (SD = ± 0.34 °C) and − 0.91 °C (SD = ± 0.28 °C). The data of every single participant is reported in Table 6.

**Table 6:** Summary of all participants’ data.

Subject	CPL + (°C)	CPL − (°C)
1	0.60	− 0.80
2	0.56	− 1.46
3	0.78	− 1.36
4	0.44	− 1.23
5	1.39	− 0.80
6	0.48	− 0.69
7	1.21	− 1.40
8	0.46	− 0.89
9	1.01	− 0.82
10	0.92	− 0.42
11	0.48	− 0.72
12	0.81	− 0.70
13	0.96	− 0.91
14	0.36	− 0.50
15	0.77	− 1.01
16	0.82	− 0.83
17	0.79	− 0.66
18	0.88	− 0.95
19	0.58	− 0.55
20	0.31	− 0.83
21	1.85	− 1.39
22	0.47	− 0.80
23	1.05	− 1.02
24	0.84	− 0.95
25	1.13	− 1.21
26	0.84	− 0.74
MEAN	0.80	− 0.91
SD	0.34	0.28

*CPL +* positive ceiling performance level (100% of accuracy), *CPL −* negative ceiling performance level (100% of accuracy).

Regarding the data about skin temperature, we did not identify any relevant variation of it during the experiment. On average, the temperature measured on the right upper arm was 30.01 °C (SD = ± 0.13 °C), on the left chest was 30.86 °C (SD =  ± 0.16 °C), on the right anterior thigh was 30.26 °C (SD =  ± 0.17 °C), and on the right calf was 30.89 °C (SD =  ± 0.16 °C) (more data on Supplementary Table S1). Moreover, the mean skin temperature calculated by means of Ramanathan's Equations^21^ (T_mean_ = 0.3 × (T_chest_ + T_arm_) + 0.2 ×  (T_thigh_ + T_leg_)) was 30.47 °C (SD =  ± 0.81 °C). At the same time, the estimated core temperature measured on the forehead of the participants remained constant at an average temperature of 36.30 °C (SD =  ± 0.11 °C) (more data in Supplementary Table S2).

## Discussion

In this study, we examined human sensitivity to environmental temperatures using a unique experimental paradigm. By having participants move between climate chambers (Fig. 2) with temperatures ranging from 23 to 25 °C (Fig. 1), we aimed to assess their ability to perceive temperature differences within that comfortable range. We conducted experiments using temperatures defined as comfortable according to the ANSI/ASHRAE standards (2017) to mitigate potential confounding factors related to uncomfortable temperatures. One potential confounding factor arises from Weber’s rule, which suggests that the perceived sensitivity is influenced by the initial magnitude of the stimulus^22^. For instance, when detecting a change in weight, we can perceive a difference if it shifts from 1 to 1.5 kg. However, if the weight initially stands at 50 kg and then changes to 50.5 kg, we would be unable to detect the difference, although the absolute change in weight remains the same in both scenarios. Therefore, since a similar behavioural pattern in relation to thermal stimuli may be anticipated, we opted for testing comfortable temperatures.

Furthermore, another potential confounding factor that may arise from testing uncomfortable temperatures is associated with the concept of alliesthesia, as originally defined by Cabanac (1971). Alliesthesia refers to the phenomenon wherein the pleasantness of a stimulus depends on the internal state of the observer^23^. In the context of thermal stimuli, this implies that the perception of a specific temperature can vary based on the previous condition of the observer. For example, individuals undergoing hyperthermia might find the same cool temperature more agreeable than if they were initially exposed to a neutral or even colder temperature^5^. Parkinson and colleagues (2016) provided evidence of this phenomenon through an experiment involving participants exposed to different temperature ramps simulating the experience of entering or exiting a fresh indoor environment in summer and a warm one in winter. Participants were asked to report their whole-body thermal sensations before and after each ramp. The results revealed that a single temperature can evoke either a pleasurable or unpleasurable response contingent on its capacity to restore participants’ comfort. Specifically, a temperature of 20 °C was positively perceived when transitioning from an environment with 30 °C (simulating the summer scenario of entering a fresh indoor ambient), while 30 °C became more positively perceived than 20 °C when preceded by an ambient temperature of 20 °C (simulating the winter scenario of entering a warm indoor ambient)^24^. Such findings underscore the potential variability that both uncomfortable temperatures and the prior internal state of participants introduce to the realm of thermal perception. Given the pioneering nature of our investigation, we deliberately opted for testing a neutral thermal condition where such phenomenon is less likely to manifest. Additionally, participants were instructed to rest in the airlock (Fig. 2) for a duration of 5 min prior to the start of the experiment, aiming at mitigating potential confounding influences.

The results within the assessed temperature range indicate a pronounced level of sensitivity among participants, as shown by the JND of 0.38 °C (SE = ± 0.02 °C), the JND^95^ of 0.92 °C (SE = ± 0.05 °C) (see Fig. 3) and the ceiling performance levels of + 0.80 °C (SD =  ± 0.34 °C) and − 0.91 °C (SD =  ± 0.28 °C) (see Table 6). This ability was consistently demonstrated by all participants, regardless of their individual temperature preferences, suggesting that this mechanism could be automatic and intrinsic in the human body. While the JND remains a conventional psychophysics parameter, our analysis has been broadened to incorporate the JND^95^ (related to two standard deviations) and the ceiling performance levels (CPLs). Specifically, the JND and JND^95^ delineate the minimum difference that participants can perceive with 75% and 95% accuracy respectively, and their estimation has been carried out via the GLMM. The two values exhibit substantial disparity, with the minimum difference needed to correctly detect a temperature change 95% of the time being more than double with respect to temperature changes needed to achieve 75% of accuracy (JND = 0.38 °C versus JND^95^ = 0.92 °C). This discrepancy aligns with the shape of the psychometric function (Fig. 3), which shows an asymptotic trend at its endpoints. This indicates that the flatter the curve, the greater the temperature change needed for a marginal increase in probability. Nonetheless, the JND^95^ is useful to make an accurate comparison with the CPLs, denoting the minimum temperature change detectable 100% of the time—an aspect directly deduced from the raw data. These values of positive and negative CPL are aligned with the predicted JND^95^ (maximum deviation of 13%), despite the JND’s inability to capture the asymmetry between hot and cold CPLs, which is going to be further analysed in the following discussion. Given the potential real-world implications of these findings, the relevance of the JND^95^ and CPLs becomes paramount, as they establish the boundaries between a temperature change that may or may not be perceived by participants.

The importance of analysing our data in light of the JND^95^ and CPLs is closely intertwined with the potential applicability of the findings. Our results hold the potential to furnish novel insights into addressing the complex challenge of achieving optimal temperature regulation within architectural structures. Traditionally, this challenge has been the focus of investigation by engineers^25,26^. Our perspective, grounded in cognitive psychology, aims to delve into the complexities of human thermal perception and harness its unique attributes. Thus, it appears pivotal to explore the threshold at which individuals marginally detect temperature shifts, as exemplified by the JND^95^. This knowledge could represent a new dowel in the spectrum of factors important to determining the optimal temperature that is not only acceptable to building occupants but also contributes to energy conservation efforts^27,28^. Simultaneously, the observations derived from CPLs, which highlight an asymmetry by revealing heightened sensitivity to warmth, hold the potential in informing the development of intelligent algorithms. These algorithms could differentially regulate temperature during summer and winter months. To better understand the possible differences between the two seasons, future research may explore potential alterations in thermal sensitivity when subjected to both colder and hotter temperature conditions.

Interestingly, our findings contrast with a previous study by Crucianelli et al. (2021) that reported larger thresholds for perceiving temperature differences on specific body parts. In particular, they found a thermal threshold for cold stimuli applied to the palm of the hand and to the forearm of respectively 2.02 °C and 1.48 °C. At the same time, they reported the threshold for warm stimuli applied in the same body part of 2.65 °C and 2.61 °C. Crucianelli and colleagues employed an adaptation of the Martsock methods of limits^29^, which involves a continuous rate of temperature change (1 °C/s). Our study, instead, involved discrete changes as participants moved between chambers. The greater ability to detect discrete changes than continuous ones^30^ may address the difference between our results and previous ones.

Another possible explanation is that in Crucianelli and colleagues’ study, the thermal stimuli were directly applied to specific parts of the participants’ bodies. In contrast, in our experiment the entire body was exposed to the thermal stimuli (i.e., the air of the chambers), allowing for a more widespread and generalised contact with the temperature variations. It is plausible that when the whole body or specific body parts are exposed to thermal stimuli, such as ambient temperature, the brain integrates these sensations from various sources and combines them to form an overall perception of the temperature. This integration of thermal signals from multiple areas of the body may enhance the sensitivity to temperature changes, leading to a better overall perception of temperature differences. Considering the complexity of thermal perception and the involvement of various neural pathways, it is likely that both the direct stimulation of specific body parts and the overall exposure of the body to temperature variations contribute to our ability to perceive and discriminate different temperatures. Supporting this hypothesis, Defrin et al. (2009) presented evidence that thermal perception can be contingent upon spatial summation. Specifically, they observed that thermal thresholds for both warm and cold stimuli increased as the stimulation area decreased, indicating that larger portions of the skin surface contribute to enhanced sensitivities^31^. Moreover, throughout the course of our experiment, participants wore t-shirts that left their arms exposed, while the rest of their bodies remained covered. Our selection of clothing was made in light of the well-documented evidence indicating that the head and hands exhibit significantly greater sensitivity compared to the legs^32–34^. This approach ensured that the more receptive regions, namely the head and hands, were left uncovered, while the remaining areas were clothed. Consequently, it is conceivable that participants’ sensitivity might have been increased as a result. Further investigations are needed to unravel the specific mechanisms involved in the integration of thermal signals and how different body parts contribute to the perception of environmental temperatures.

As already mentioned, we observed a marginally more precise ceiling performance level for warmth (CPL = + 0.81 °C) compared to cold (CPL = − 0.91 °C), indicating that participants displayed a higher sensitivity to warmth. It is important to highlight that our experiment was designed to avoid any asymmetry in the experienced temperature differentials between the chambers. This was achieved by implementing an oscillating temperature range of ± 1 °C around 24 °C within each chamber (Fig. 1). The extensive dataset encompassed 1568 trials with positive temperature differences and 1552 trials with negative temperature differences. Exploring existing literature, apart from the head—known to be more responsive to warm stimuli than cold^6^—other body regions typically exhibit greater sensitivity to cold stimuli^19,35^. Given that participants had exposed arms, their overall thermal perception likely arose from a combination of sensations originating from both the arms and the head. Consequently, attributing the marginally more accurate CPL for warm stimuli to the increased sensitivity of the head becomes challenging. A potential explanation might be linked to participants’ tendency to more frequently report the target chamber as warmer, as evidenced by the negative PSE (− 0.13 °C). Such a tendency could potentially culminate in a reduced CPL, as participants would exhibit fewer errors when the genuinely warmer chamber is identified. Importantly, this does not inherently signify a higher sensitivity to warmth, but rather a perceptual bias influenced by participants’ response patterns.

In line with this hypothesis, we unexpectedly found a negative point of subjective equality (PSE) of − 0.13 °C, which can be approximated to 0 °C considering the standard deviation (± 0.12 °C) and sensor uncertainty (± 0.06 °C). Given that the tested temperatures were within the comfortable range according to ANSI/ASHRAE standards (2017), it was anticipated that participants would not feel warm or cold while still in the air-lock. Additionally, half of our participants are individuals who typically experience rapid sensations of warmth and exhibit a preference for colder temperatures. Notably, only in this part of the population, which is more susceptible to feeling warm, we could have anticipated a negative PSE indicating a greater proportion of “warmer” answers. Nevertheless, on average, all our participants showed this behaviour, consistently perceiving the target chamber as warmer compared to the reference chamber. While the participant pool consisted of only a limited number who explicitly preferred warmer temperatures, it is noteworthy that among the nine participants who displayed no distinct prior temperature preferences, eight exhibited a negative PSE. This finding indicates that the distribution of temperature preference among participants did not significantly affect the overall results. To delve deeper into this aspect, we conducted our analysis using GLMM, with participants as a random effect. This step was taken to assess whether participants’ responses introduced noteworthy variability in the data. The analysis revealed that when considered in isolation, the random effect failed to yield a significant explanatory impact on the data (R2m = 68% versus R2c = 72%). This additional analysis further reinforces the conclusion that participants did not substantially contribute to data variability. As another possible explanation, we also considered that the act of moving between chambers and performing the task might have increased participants’ body temperature. The physical activity involved in walking could have resulted in a temporary increase in body heat, causing participants to subjectively perceive the target chamber as warmer. Nonetheless, accounting for the average time of 15 s required for participants to cover the average distance of 7.5 m between the reference chamber and the target chamber during each transition (Fig. 2), we can deduce an estimated Metabolic Equivalent of Task (MET) value of 3. Considering that the threshold for moderate activity is 4.9 METs^36^, we may conclude that our task did not imply a relevant increase in body temperature. As additional proof, no relevant variations in the local skin temperature and in the core temperature were identified (see Table S1 and S2). It remains thus unclear why a negative PSE was observed.

Despite this unexpected finding, our study has important implications for real-life applications, particularly in indoor environments. By demonstrating participants’ limited capacity to discern temperature differences of less than ± 0.92 °C, as shown by the JND^95^, our results offer valuable insights with implications for energy-conservation strategies. Optimising indoor temperature control algorithms by adjusting the temperature within the imperceptible range in either direction during summer and winter, could lead to significant energy savings and cost reductions without compromising occupant comfort.

## Conclusions

Our study employed a novel experimental paradigm, involving 26 participants, to investigate human sensitivity to environmental temperatures. The findings revealed that participants exhibited remarkable accuracy in detecting temperature changes, as shown by the JND of 0.38 °C, the JND^95^ of 0.92 °C and the CPLs of + 0.8 °C and − 0.91 °C. This threshold appears to be smaller than those identified for specific body parts in previous studies, indicating the need for further investigation into this discrepancy. Moreover, the limited variability in participants’ behaviours, as indicated by the small difference between the marginal and conditional R-squared (R2m = 0.68, R2c = 0.72), suggests that the mechanism underlying thermal sensitivity could be automatic and intrinsic to every individual’s body. These findings contribute to our understanding of human temperature perception and may have practical implications for energy-efficient temperature control in indoor environments.

## Methods

### Participants

A total of 26 participants (13 males and 13 females) took part in our research. Table 1 summarises the characteristics of the participants. A priori power analysis and a review of the literature^37^ indicated that our sample size provided sufficient power (0.8) to detect the effects of interests. Inclusion criteria for participants comprised being between 18 and 65 years old, having a BMI in the range of 18.5–24.9, and being able to provide informed content. Exclusion criteria consisted of having a history of psychiatric or neurological conditions, a cardiac illness, health or sensory conditions that might result in skin alterations (e.g., psoriasis), and suffering from claustrophobia. The study was approved both by the ethical committee of the University of Trento and by the Azienda Sanitaria of the province of Bozen, and was conducted in accordance with the Declaration of Helsinki (Fortaleza 2013). Moreover, all participants gave their informed consent prior to starting the experiment.

We administered an online questionnaire to assess participants' eligibility and gather information about relevant psychological aspects. The questionnaire included the following measures:

1. Experienced Temperature Sensitivity and Regulation Survey (ETSRS^38^): This survey was used to determine participants’ usual temperature preferences. Employing two distinct ordinal scales, each comprising 7 points, the first ranging from “Much cooler” to “Much warmer”, and the second spanning from “Much later” to “Much quicker”, participants were categorised as follows: those favouring cold temperatures (with a predominance of “much cooler” responses for temperature preference and “much quicker” for the speed of experiencing warmth), those preferring warmer temperatures (characterised by a predominance of “much warmer” responses for temperature preference and “much later” for the speed of experiencing warmth), and those without specific temperature inclinations.
2. Empathy Quotient (EQ^39^): The EQ was used to assess participants’ level of empathy by means of a 4-point Likert scale going from “Strongly agree” to “Strongly disagree”. Empathy has been shown to affect activity in the somatosensory cortex, which is relevant to the processing of thermal stimuli^40^.
3. Body Perception Questionnaire (BPQ^41^): The BPQ was used to evaluate participants’ level of awareness of their own body’s internal states. It uses a 3-point Likert scale going from “Never” to “Often”.
4. Global Physical Activity Questionnaire (GPAQ^42^): We employed the GPAQ to gather information about participants’ typical level of daily physical activity. This was relevant as our task involved periods of movement lasting 15 min.

Throughout the experiment, participants wore standardised clothing (0.5 clo) to ensure consistent skin coverage and equal thermal insulation between their bodies and the environment. Both male and female participants wore long jeans, short-sleeved t-shirts, and closed shoes. To monitor the participants’ skin temperature, we placed four thermal sensors (SHT31 Smart Gadget from Sensirion company) in specific locations on their bodies: the left part of the chest, the right upper arm, the right anterior thigh and the right anterior calf. We based this decision on a previous work of Liu and colleagues^43^, who have shown that using four sensors in these sites reliably assesses the mean skin temperature of the body. Additionally, we measured participants’ forehead temperature as an estimate of their core temperature at the beginning, midpoint, and conclusion of the experiment using an infrared laser thermometer (model UC-03A from Yiercom company) (results reported in Supplementary Table S2). The core temperature refers to the temperature of the internal organs and is commonly evaluated through invasive methods, such as pulmonary artery catheters or esophageal probes^44^. Nevertheless, infrared laser thermometers directed at the forehead have demonstrated satisfactory reliability as a means to measure an approximate body core temperature^45^.

### Apparatus

We used four different climate chambers connected through an air-lock (Fig. 2), used as a waiting room before the test and between the different experimental blocks. The temperature in the airlock was monitored throughout the entire test, with a mean value of 22.8 °C (see Supplementary Fig. S2). The four chambers (3 m × 3 m × 3 m each) were kept at a constant relative humidity equal to 45% while the temperatures were oscillating between 23 and 25 °C as shown in Fig. 1. The temperature range was chosen in the comfort range to prevent some possible confounds that could emerge from an uncomfortable condition. We defined these temperatures as comfortable by looking at the ASHRAE standards (ANSI/ASHRAE, 2017) and by conducting a pilot. In order to evaluate the thermal stratification during the experiment, three PT100 temperature sensors (uncertainty of ± 0.06 °C, k = 2) were placed at three different heights corresponding to the head, the arm and the calf of the participant. The effect of the thermal stratification is shown in Fig. S1. We chose as temperature reference the average temperature measured at the head and arm level given the exposed participant’s skin due to the clothing. During the whole experimental campaign, the chambers’ temperature pattern was kept the same (Fig. 1), while each participant’s shift pattern between the chambers was randomised. Our aim was to prevent participants from developing a cognitive schema for detecting the temperature pattern and relying on it to make judgments about temperature differences between the chambers. To achieve this, we designed the temperature fluctuations to follow a complex pattern that would be difficult for participants to discern, thereby forcing them to rely solely on their sensory experience to make temperature judgments. This approach minimised the risk of bias in participant responses and ensured that the data collected was an accurate reflection of their perceptual experiences.

### Procedure

Throughout the experiment, participants transitioned between different climate chambers and compared the temperature of the target chamber (the second they moved in) to that of the reference chamber (the first one they entered). We maintained stable communication with the participants using walkie-talkies, and we monitored the entire process using five video cameras. Participants were allowed to spend 5 s in each chamber (both reference and target) to perceive and assess the temperature difference. The decision to employ a 5-s time interval was based on a pilot study, where we determined that this duration was most effective in guaranteeing participants’ discernment of thermal stimuli. Additionally, this interval allowed us to maintain the overall experimental procedure within a duration shorter than two hours, mitigating the potential for participants' fatigue. During the permanence time, participants stood still in the middle of the chambers and were free to keep their eyes closed/open. Upon leaving the target chamber, participants provided their responses in the airlock area. Each experimental block consisted of 24 temperature comparisons and lasted approximately 15 min. There were a total of 5 experimental blocks, with 5-min breaks between them to allow participants to rest. Overall, the entire procedure took 100 min to complete, involving a total of 120 temperature comparisons.

### Analysis

**Figure 4:**
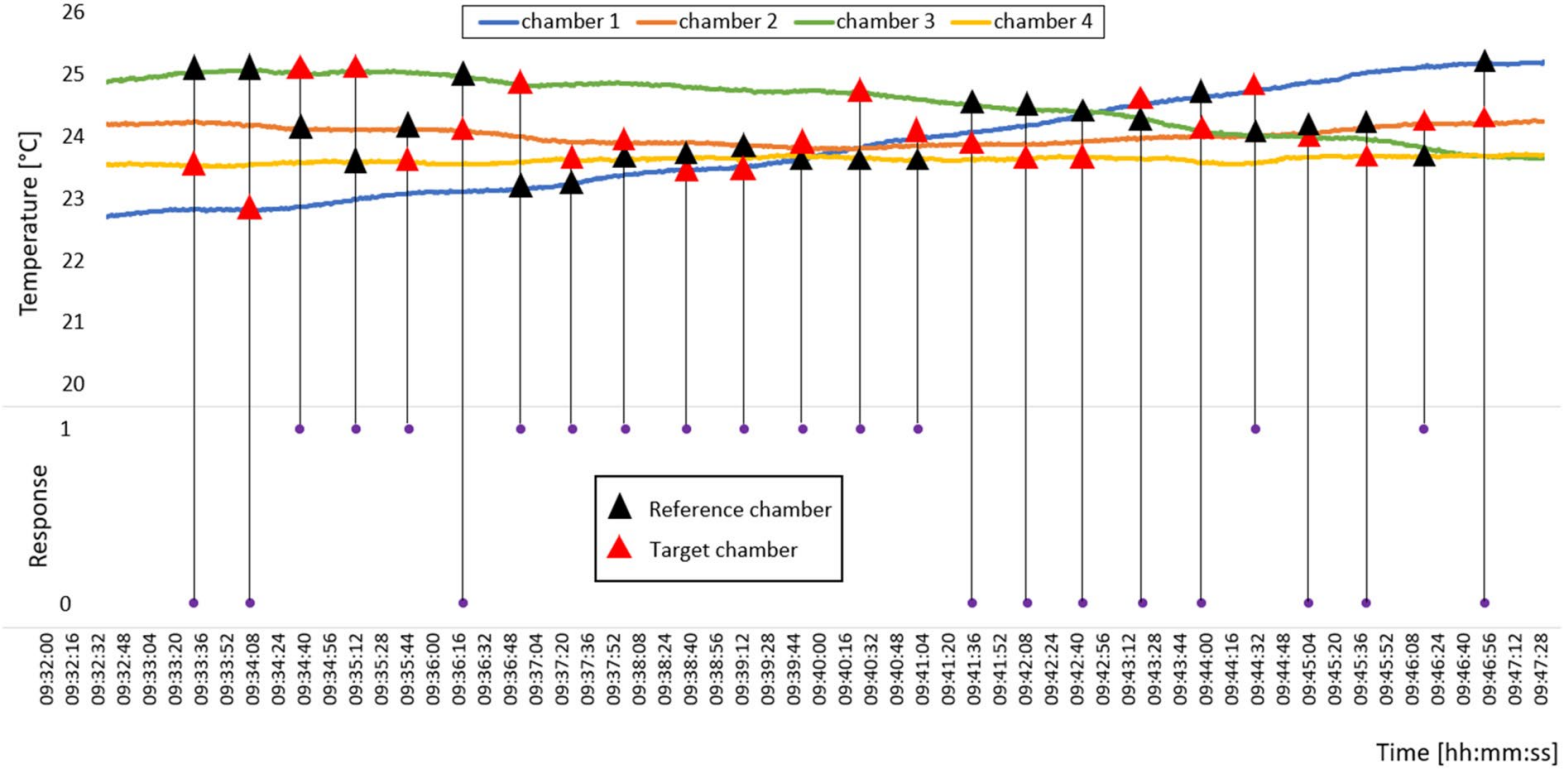
Example of temperature patterns in the four climate chambers and of the participant’s answers during one experimental block.

Figure 4 reports the example of one experimental block with the temperature variations inside the four climate chambers and the 24 comparisons the participant made together with the answers. For each comparison, we measured the temperature difference between the target chamber (red triangle), i.e. the second chamber participant entered, and the reference chamber (black triangle), i.e. the first chamber participant entered. Then, we compared each temperature difference with the participant’s answer, where 1 means “warmer” while 0 means “colder”. We grouped the data obtaining a final dataset of 3120 observations in 3 variables (subject, temperature difference and answer).

Next, we fitted two generalised linear mixed-effects models^46^ using the function glmer inside RStudio (version 2022.02.0 + 443). Given that we computed repetitive measures on single participants, we decided to analyse our data using generalised linear mixed-effects models that better take into account such sources of variability^47^. The first model we computed contained the number of colder and warmer answers and the differences in temperature as fixed effects and the subjects ID as a random effect (glmm0). The second model added to the previous one the difference in temperature as a random effect (glmm1) to see whether this aspect of our experiment brought variability in the data. Then we compared the two models through means of an ANOVA and looked at which model had the smallest AIC criterion. Finally, we computed the marginal and conditional R^2^ of the best model to analyse the percentage of variance explained respectively by the fixed effects only and by the fixed effects plus the random ones.

Then, we calculated the average Point of Subjective Equality (PSE) and the Just Noticeable Difference (JND) using the MixDelta function in R^48^. Moreover, we calculated the JND with respect to two standard deviations (i.e., representing 95% of accuracy, further indicated with JND^95^) to compare it with the ceiling performance levels (CPL). The CPLs represent the minimum value needed to correctly identify the difference in the temperature 100% of the time. To obtain this data, for each participant we looked at the positive difference in temperature above which all answers were “warmer” (positive CPL) and the negative difference in temperature below which all answers were “colder” (negative CPL). Finally, we obtained the general CPLs by averaging these data for all the participants.

### Supplementary Information

Supplementary Information.

## Data Availability

Anonymous data and analysis script can be shared on request by contacting the first author Laura Battistel at her personal email address: laura.battistel@unitn.it.
