# Author Correction: An investigation on humans’ sensitivity to environmental temperature

**DOI:** 10.1038/s41598-023-50297-9

**Published:** 2023-12-21

**Authors:** Laura Battistel, Andrea Vilardi, Massimiliano Zampini, Riccardo Parin

**Affiliations:** 1 Center for Mind/Brain Sciences (CIMeC), University of Trento, Corso Bettini 31, Rovereto, TN Italy; 2 terraXcube, Eurac Research, Via Ipazia 2, 39100 Bolzano, Italy

Correction to: *Scientific Reports* 10.1038/s41598-023-47880-5, published online 04 December 2023

The Acknowledgements section in the original version of this Article was incomplete. It now reads:

“The authors thank the Department of Innovation, Research University and Museums of the Autonomous Province of Bozen/Bolzano for covering the Open Access publication costs. The authors are grateful to all the participants involved in the study and to the terraXcube team, which helped to run the chambers smoothly. Moreover, the authors thank Claudio Zandonella Callegher and Alice Deruti for their contribution given to the data analysis.”

The original Article has been corrected.

