# Supplementary material for: An investigation on humans’ sensitivity to environmental temperature
Source: Sci Rep. 2023 Dec 4;13:21353. doi: 10.1038/s41598-023-47880-5 (PMC10695924; doi:10.1038/s41598-023-47880-5)
Supplement: Supplementary file 1 — Supplementary Information. [file 41598_2023_47880_MOESM1_ESM.pdf]

Supplementary Materials

Figure S1 shows an example of space and time temperature distribution during the entire experiment in one chamber. In particular, it can be seen how in the low part of the chambers (foot level) there was always a lower temperature in comparison to the temperature measured at greater heights (arm and head level) due to the natural circulation effect.

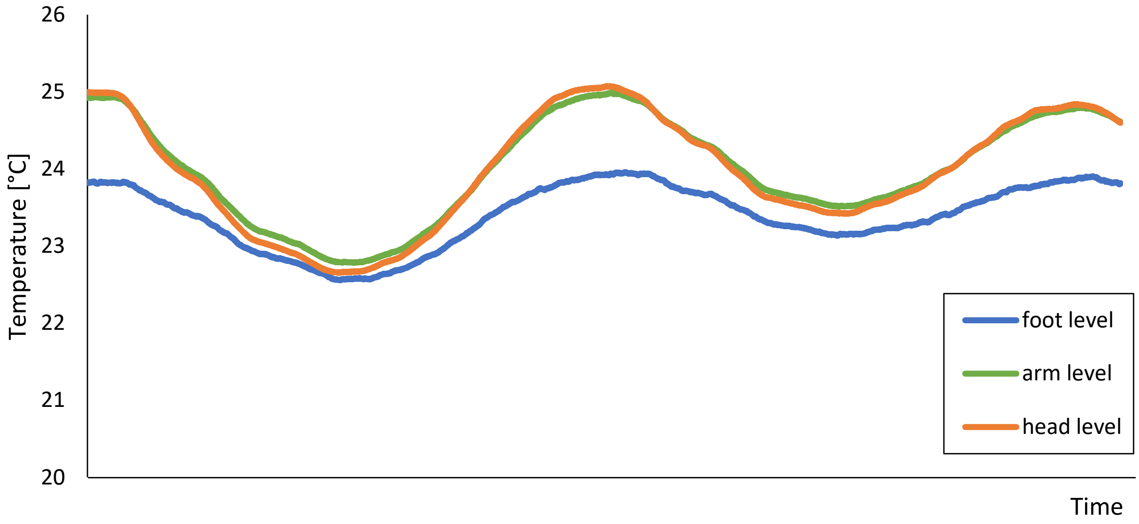

Figure S1: temperature stratification during the experiment at three different heights (foot level, arm level and head level).

Figure S2 reports the average behaviour of the temperature in the air-lock where the participants rested at the beginning of the experiment and during the breaks between the blocks.

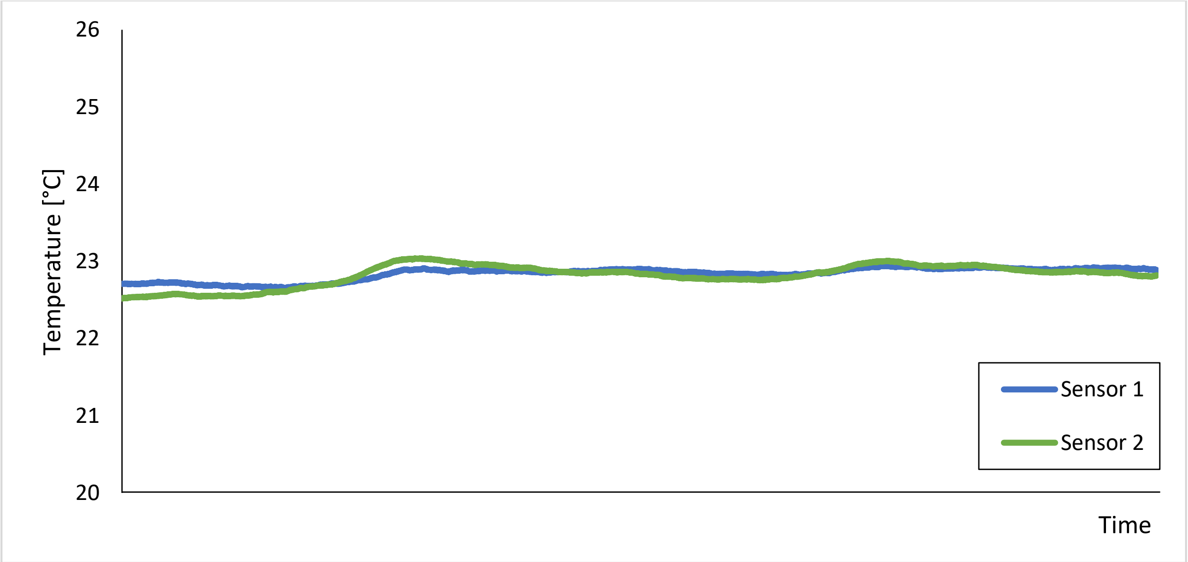

Figure S2: Temperatures of the air-lock measured during the experiment. The average temperature was of 22.8°C

Figures from S3 to S8 show an example of how the differences in the temperature of the four chambers changed during one experimental block. Moreover, Figure S9 presents the distribution of trials as a function of the differences in the temperature for one participant. More specifically, we divided the trials into those which had a difference in the temperature between 0 °C and ± 0.5 °C, a difference between ± 0.5 °C and ± 1 °C, a difference between ± 1 °C and ± 1.5 °C, a difference between ± 1.5 °C and ± 2 °C, and a difference between ± 2 °C and ± 2.5°C. This analysis shows that the majority of trials fell inside the first two intervals, i.e. the intervals in which participants had the greatest difficulty in the task.

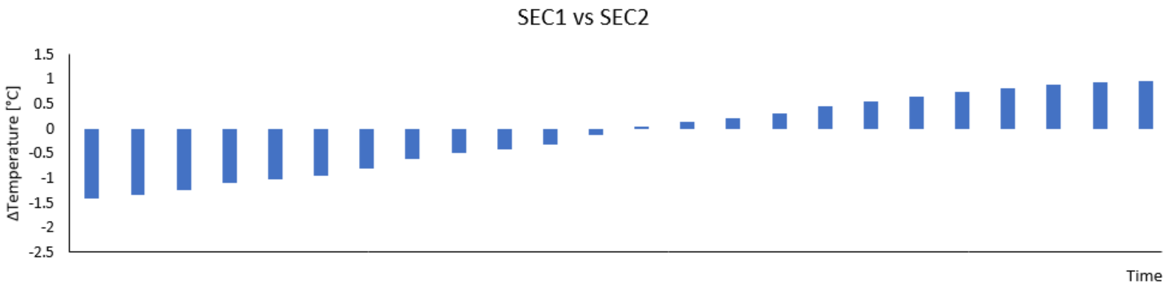

Figure S3: temperature difference between chamber 1 (SEC1) and chamber 2 (SEC2) during one experimental block

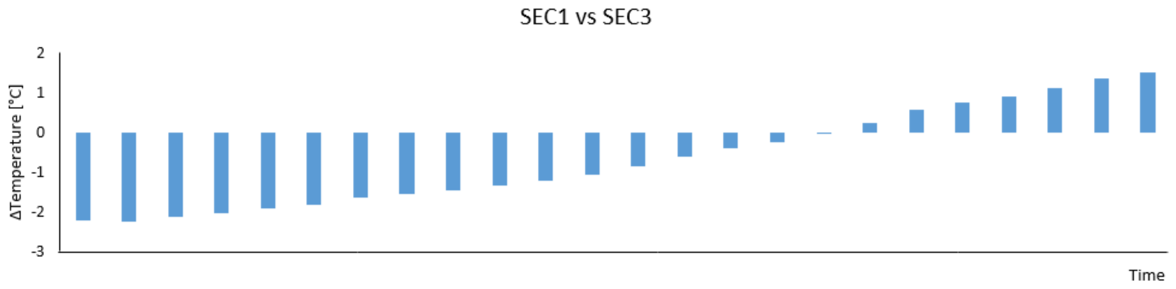

Figure S4: temperature difference between chamber 1 (SEC1) and chamber 3 (SEC3) during one experimental block

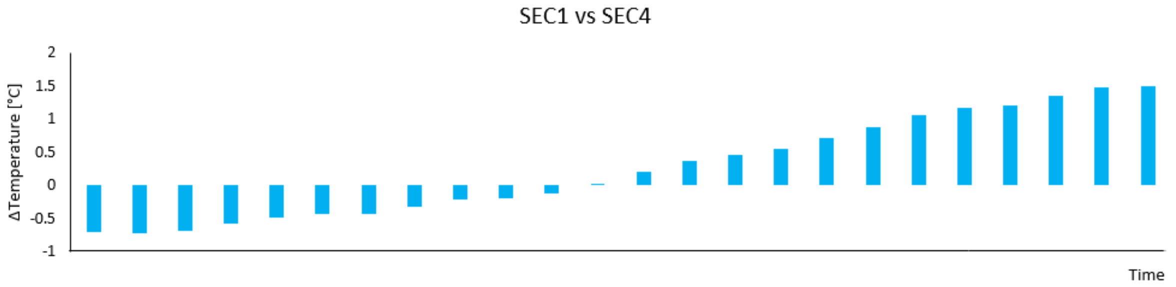

Figure S5: temperature difference between chamber 1 (SEC1) and chamber 4 (SEC4) during one experimental block

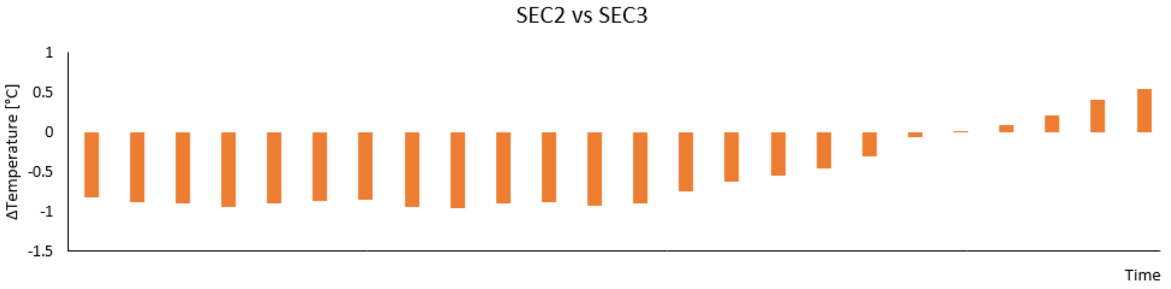

Figure S6: temperature difference between chamber 2 (SEC2) and chamber 3 (SEC3) during one experimental block

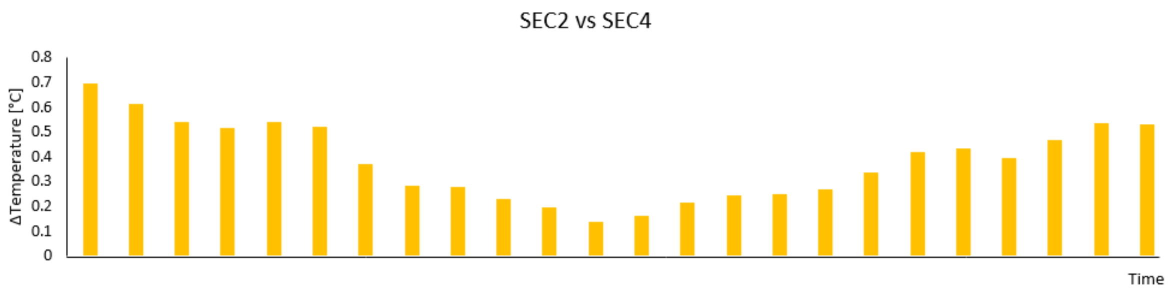

Figure S7: temperature difference between chamber 2 (SEC2) and chamber 4 (SEC4) during one experimental block

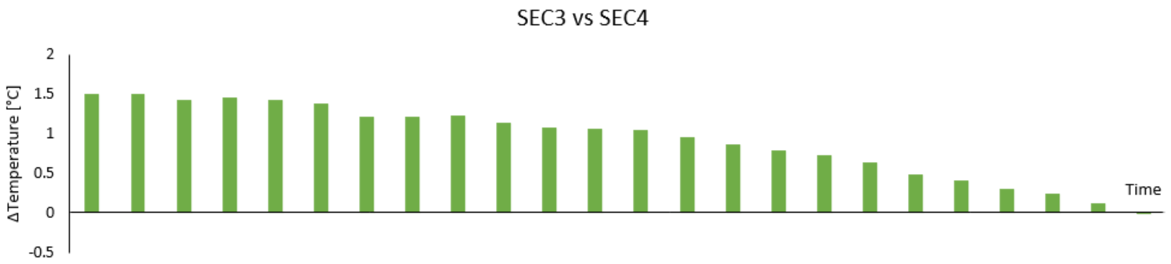

Figure S8: temperature difference between chamber 3 (SEC3) and chamber 4 (SEC4) during one experimental block

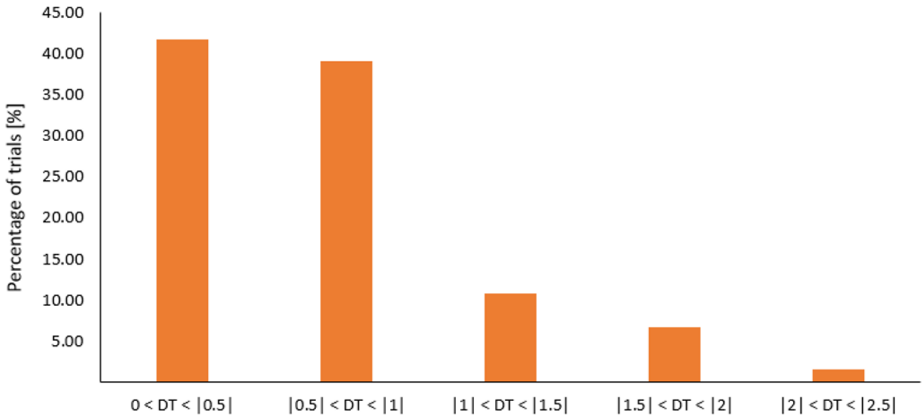

Figure S9: percentage of trials for each temperature difference divided in intervals

Table S1 presents all participants' data about skin temperature. When data is not reported, it means that the sensor(s) did not work for some unknown reason.

Table S2 shows the results of the core temperatures for all the participants. For each one of them, it is reported the mean result across the 3 measures (beginning, middle, and end of the experiment). It can be noticed, that the temperature measured from the forehead on average is 36.3 °C and very stable among all the participants (SD = ± 0.11 °C).

	S. Arm		S. Chest		S. Thigh		S. Calf	
	Mean	SD	Mean	SD	Mean	SD	Mean	SD
P1	30.92	0.36	31.25	0.49	31.11	0.75	30.22	0.58
P2	30.38	0.22			31.54	0.87		
P3	31.40	0.27	30.17	0.84	31.27	0.40	31.33	0.42
P4	29.69	0.33	29.67	0.38	29.92	0.53	32.73	0.42
P5	30.97	0.67	32.85	0.22	30.47	0.55	31.88	0.61
P6			31.44	0.31			31.31	0.78
P7			30.11	0.29	30.32	0.54	29.71	0.33
P8	30.63	0.33	30.24	0.27	31.04	0.58	32.39	0.38
P9	29.38	0.21	29.42	0.29	30.25	0.64	30.51	0.68
P10	30.12	0.27	31.22	0.64	30.92	0.33		
P11			32.67	0.45	30.85	0.61		
P12	31.17	0.44	31.41	0.34	31.44	0.61	29.92	0.43
P13	29.95	0.26	31.13	0.35	30.72	0.81		
P14	29.59	0.28	31.47	0.23	29.82	0.16	31.55	0.62
P15	27.60	0.19	30.15	0.41	27.85	0.84	29.18	0.72
P16			31.20	0.42			31.26	0.42
P17			30.05	0.22			31.10	0.77
P18			29.40	0.58			30.30	0.31
P19	29.01	0.56	31.29	0.11	30.14	0.42	31.79	0.65
P20			31.06	0.38	28.74	0.54		
P21					30.95	0.69	29.70	0.50
P22	29.86	0.27	32.19	0.46	29.88	0.59		
P23			29.39	0.26	29.98	0.61		
P24			30.70	0.26			33.00	0.57
P25	29.01	0.18	31.38	0.28	28.00	0.73	29.81	0.86
P26	30.53	0.27					29.31	0.55
MEAN	30.01	0.13	30.86	0.16	30.26	0.17	30.89	0.16

Table S1: average skin temperatures measured on the four sites for the 26 participants

	S. Forehead	
	Mean	SD
P1	36.27	0.06
P2	36.20	0.10
P3	36.33	0.06
P4	36.30	0.00
P5	36.30	0.17
P6	36.17	0.12
P7	36.27	0.06
P8	36.37	0.06
P9	36.27	0.06
P10	36.37	0.21
P11	36.33	0.06
P12	36.50	0.17
P13	36.63	0.15
P14	36.27	0.06
P15	36.30	0.00
P16	36.33	0.12
P17	36.23	0.06
P18	36.23	0.06
P19	36.30	0.00
P20	36.27	0.06
P21	36.03	0.06
P22	36.33	0.15
P23	36.20	0.10
P24	36.27	0.06
P25	36.27	0.12
P26	36.40	0.00
MEAN	36.30	0.11

Table S2: average core temperatures measured on the forehead and the back of the hand for the 26 participants
